# Multifactorial Pulmonary Hypertension in Systemic Sclerosis

**DOI:** 10.7759/cureus.9144

**Published:** 2020-07-11

**Authors:** Habiba Hussain, Ronald Espinosa, Subramanyam Chittivelu

**Affiliations:** 1 Internal Medicine, University of Illinois College of Medicine, Peoria, USA; 2 Pulmonary and Critical Care Medicine, University of Illinois College of Medicine at Peoria - OSF Saint Francis Medical Center, Peoria, USA

**Keywords:** multifactorial pulmonary hypertension, scleroderma, interstitial lung disease, pulmonary arterial hypertension, systemic sclerosis

## Abstract

Pulmonary hypertension is a progressive disease often associated with multifactorial etiology. The impact of multiple causes contributing to rapid progression of the disease, to our knowledge has not been thoroughly reviewed in literature. The cause of pulmonary hypertension is often implied from pre-existing comorbidities. A diagnostic and management challenge exists when simultaneous presence of multiple plausible causes exist. Studies evaluating the rapid progression of symptoms in multifactorial pulmonary hypertension to this effect are lacking. We present a case of pulmonary arterial hypertension (PAH) in a patient with rapidly progressing symptoms to highlight the need for an early and thorough diagnostic workup.

## Introduction

The clinical presentation of pulmonary hypertension often includes exertional dyspnea and fatigue. Pulmonary hypertension may be identified as pre-capillary or post-capillary, where pre-capillary is considered as pulmonary arterial hypertension (PAH) and post-capillary hypertension may be pulmonary venous hypertension or elevation of capillary pressures. National Institute of Health (NIH) registry considers mean pulmonary arterial pressure (PAP) above 25 mmHg at rest and 30 mmHg with exertion, as diagnostic of pulmonary hypertension. The workup for PAH is extensive, including evaluation for pulmonary vascular diseases such as HIV, portal hypertension or medication induced, and necessitates right heart catheterization (RHC) for confirmation. PAH may coexist in the presence of secondary causes of pulmonary hypertension, although ascertaining the etiology of PAH may be difficult especially in late adulthood due to co-morbidities [1-3].

## Case presentation

**Figure 1:**
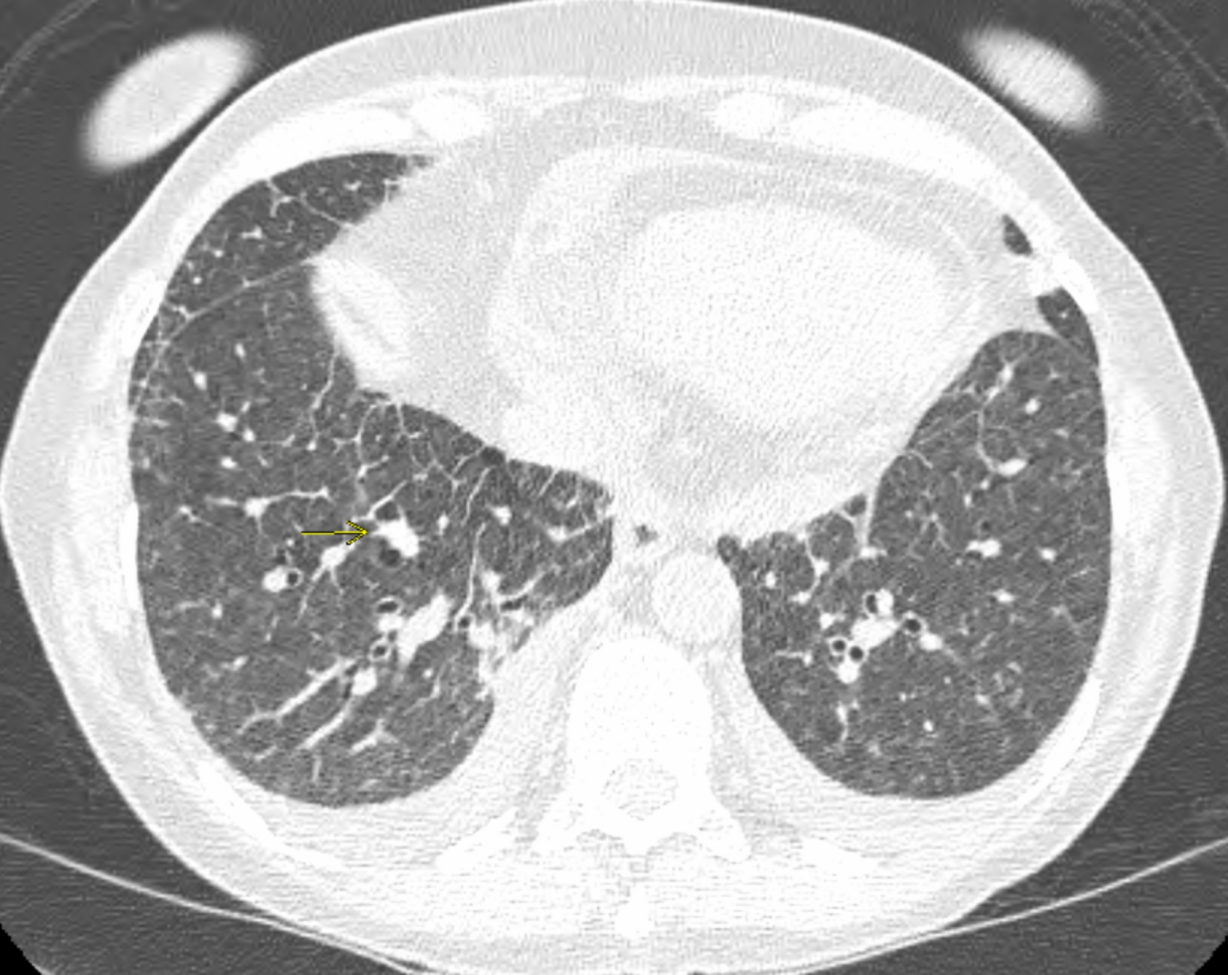
Basilar interlobular and intralobular septal thickening, ground glass opacity and unchanged pulmonary nodule.

**Figure 2:**
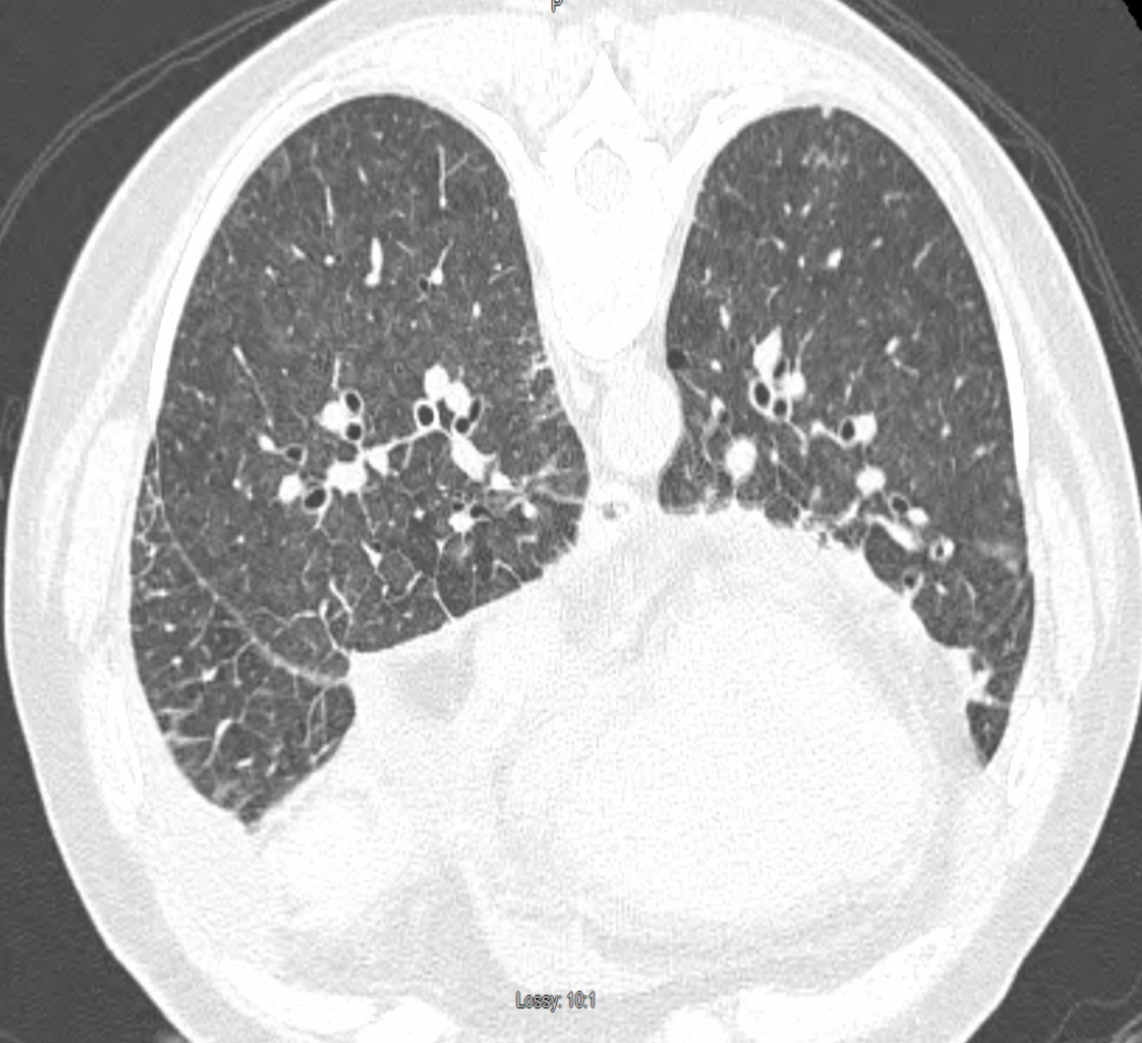
Ground glass opacity, small bilateral pleural effusions, interlobular septal thickening in the setting of pulmonary scleroderma.

A 77-year-old female with a past medical history of myelodysplastic syndrome (MDS) with 20q deletion (international prognostication score 0 - low risk) with anemia and Crohn's disease presented with complaints of nine months of dyspnea on exertion. She was on darbepoetin alfa for MDS and balsalazide for the last three years for Crohn's disease. Her symptoms had worsened recently, interfering with activities of daily living in the last few months. She reported a remote history of smoking, no association of symptoms with weather, no use of illicit drugs, anoregixens, herbal substances, etc. No personal history of clots, cardiac disease, liver disease, or family history of connective tissue disorder was noted. Examination was largely remarkable for ambulatory desaturation to 80% and bilateral rales on auscultation. She was recommended to use baseline 2 L nasal cannula oxygen due to documented desaturation with ambulation, while workup was initiated. Extensive investigations were performed with anti-nuclear antibody (ANA), antineutrophil cytoplasmic antibody (ANCA), fungal serology (histoplasma, blastomycosis, coccidiodomycosis), rheumatoid factor, anti-cyclic citrullinated peptide, micopolyspora, thermoactinovulgaris, creatinine phosphokinase (CPK), alfa1 anti-trypsin, and polysomnography. Significant results included ANA 1:640, anti-centromere antibody at > 8.0 AI, and sleep apnea requiring continuous positive airway pressure (CPAP) at 12 cm of water overnight. She was referred to rheumatology and diagnosed with systemic sclerosis (SSc) in the presence of supportive findings of Raynaud’s phenomenon, calcinosis, and telangiectasia. Pulmonary function test (PFT) showed normal pre- and post-bronchodilator forced expiratory volume in one second (FEV1) and forced vital capacity (FVC) with a ratio of 74% and 69% respectively. Diffusion capacity was decreased at 44%, with increase to 58% of predicted after correlation with alveolar volume, reflecting mild obstructive ventilatory defect. High resolution computed tomography (HRCT) showed increased ground glass and interstitial opacities in the right middle and right lower lobes (RML, RLL) (Figures 1-2).

**Figure 3:**
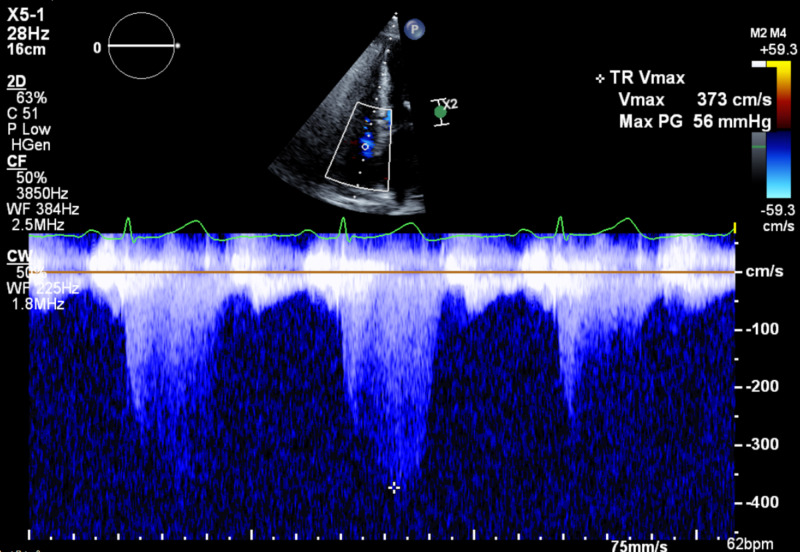
Initial TTE showing tricuspid regurgitation Vmax 373 cm/s. TTE: transthoracic echocardiogram; Vmax: velocity

**Figure 4:**
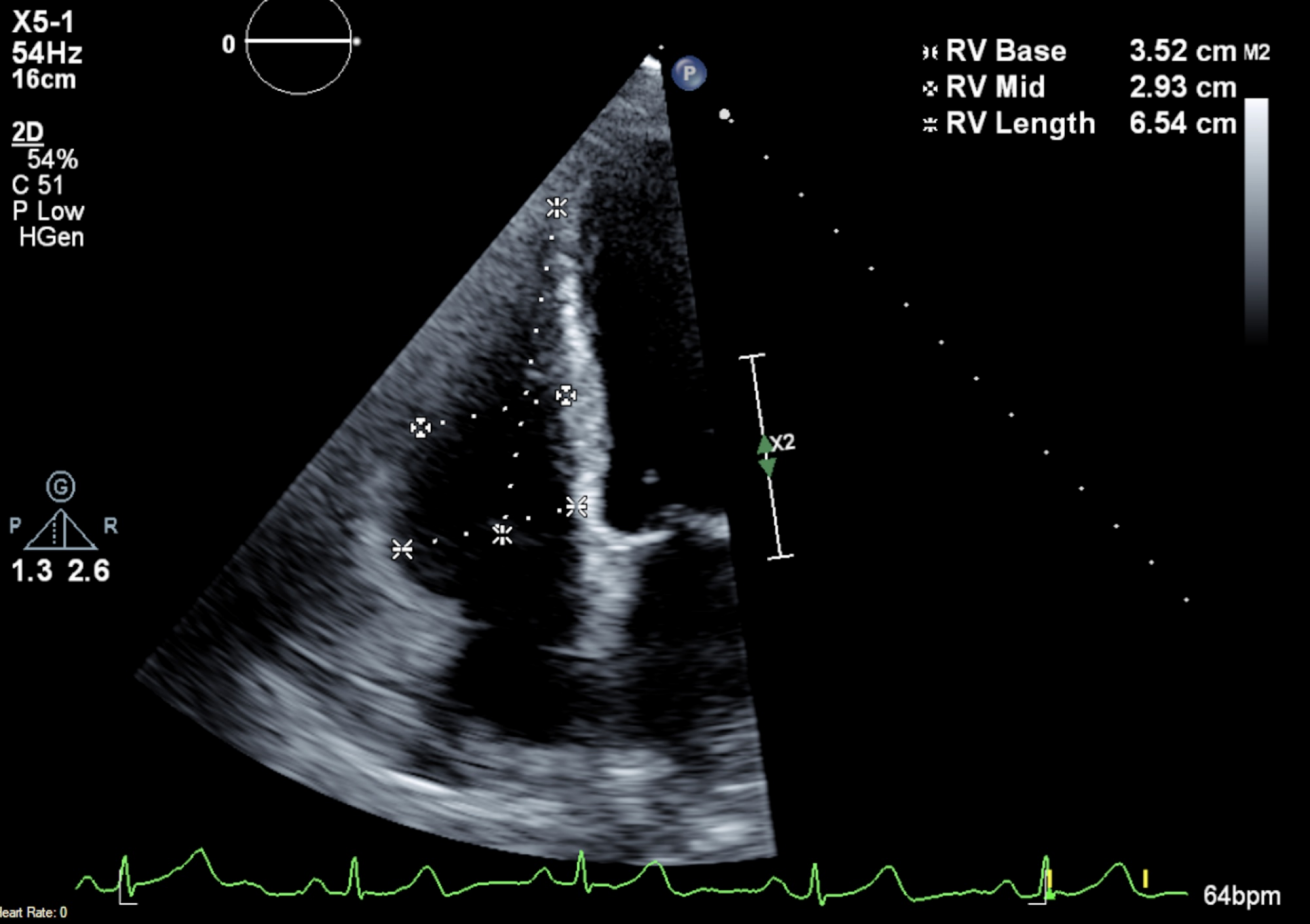
Initial TTE showing RV dimension. TTE, transthoracic echocardiogram; RV: right ventricle

**Figure 5:**
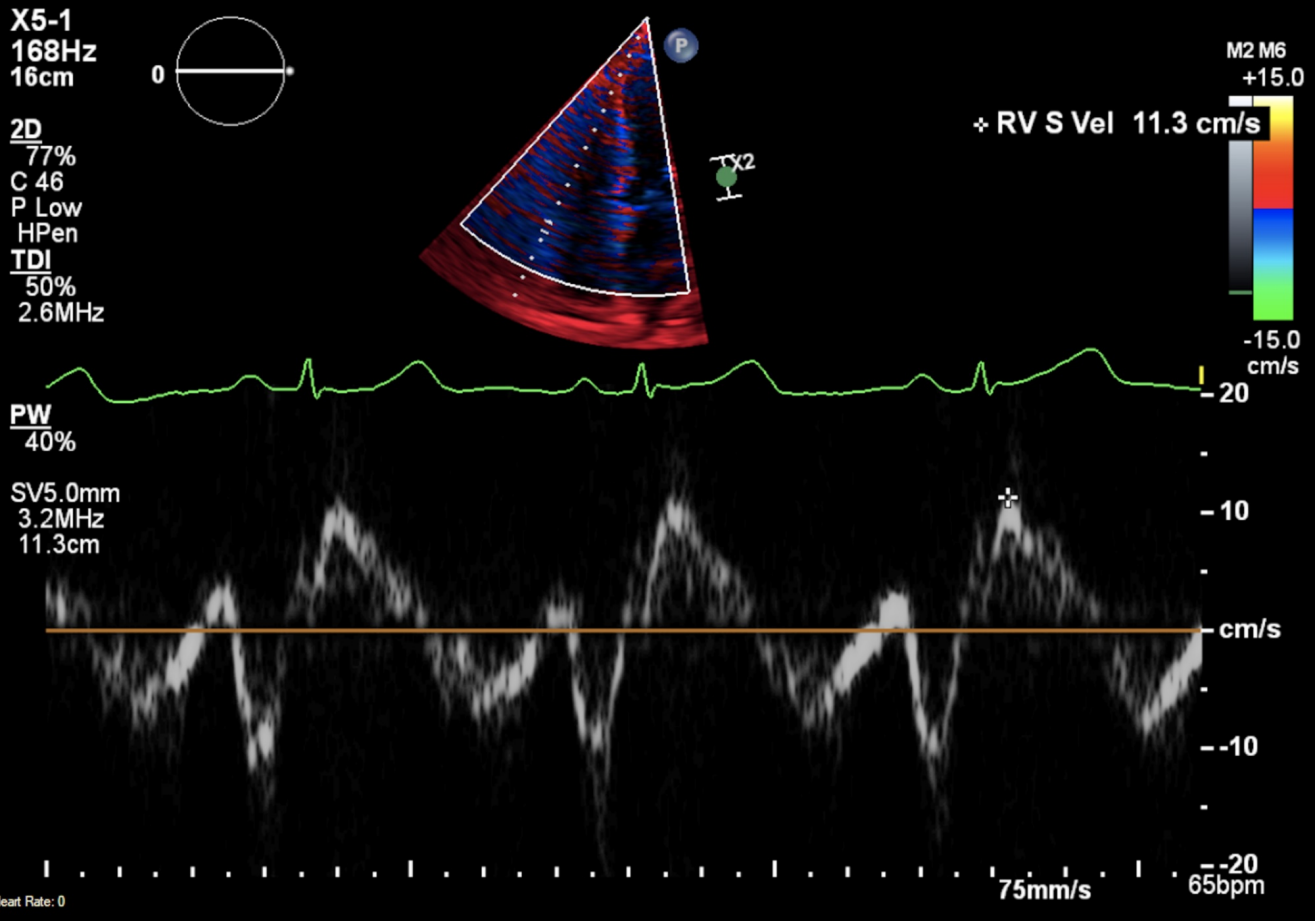
Initial TTE showing RV velocity. TTE: transthoracic echocardiogram; RV: right ventricle

Due to worsening exertion dyspnea over the next few months, repeat PFTs showed moderate obstructive disease with comparative decrease in FEV1 and FVC. Initial transthoracic echocardiogram (TTE) showed pulmonary artery systolic pressure of 59 mmHg with grade 2 diastolic dysfunction, thus confirming presence of pulmonary hypertension in the setting of SSc along with interstitial lung disease (ILD), obstructive sleep apnea (OSA), heart failure with preserved ejection fraction, MDS, and chronic anemia (Figures 3-5).

Ventilation-perfusion (V/Q) scan was also performed showing no evidence of abnormal perfusion patterns, hence ruling out chronic thromboembolic pulmonary hypertension (WHO group IV).

**Figure 6:**
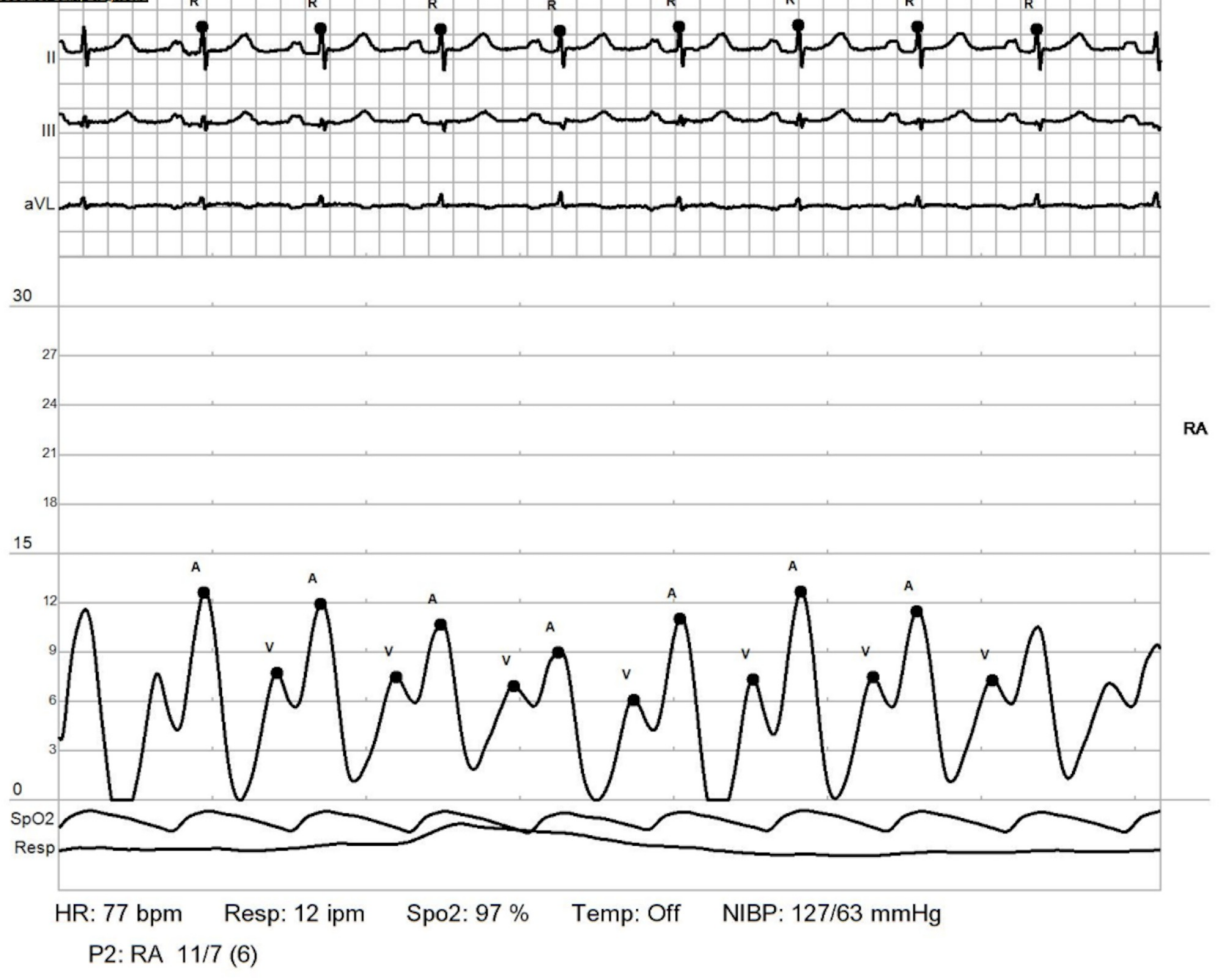
RHC: right atrium. Shows elevated right atrial pressures RHC: right heart catheterization

**Figure 7:**
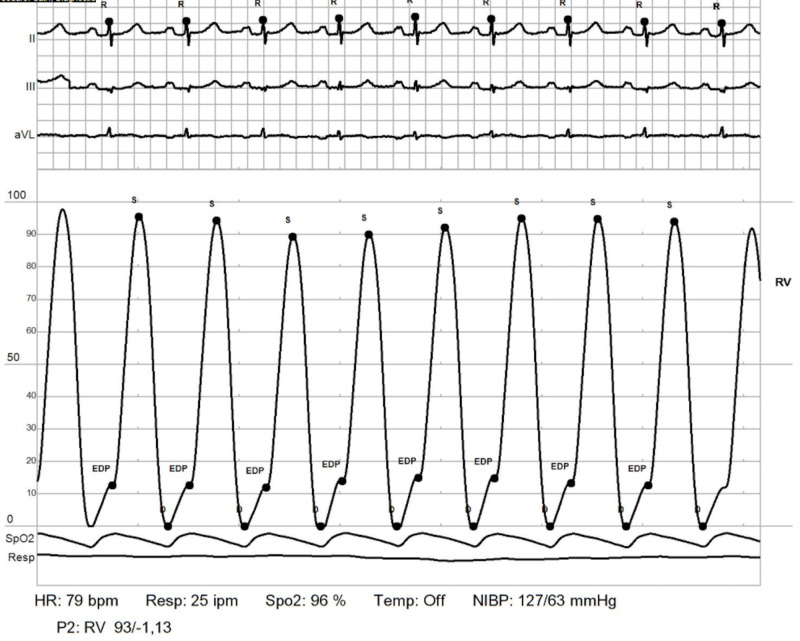
RHC: RV. Elevated RV end diastolic pressure RHC: right heart catheterization; RV: right ventricle

**Figure 8:**
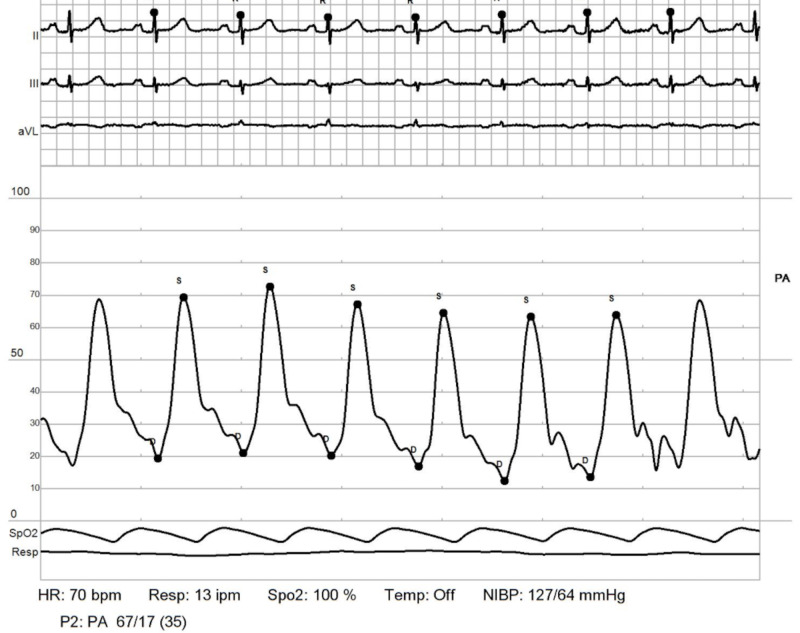
RHC: PCWP. Pulmonary arterial hypertension (PAH) as a measure of pulmonary vascular disease is indicative with pulmonary vascular resistance >3 Woods Units and an elevated transpulmonary pressure gradient (TPG) > 12 mmHg. TPG is the difference between mean pulmonary artery pressure (PAP) and PCWP. RHC: right heart catheterization; PCWP: pulmonary capillary wedge pressure;

**Figure 9:**
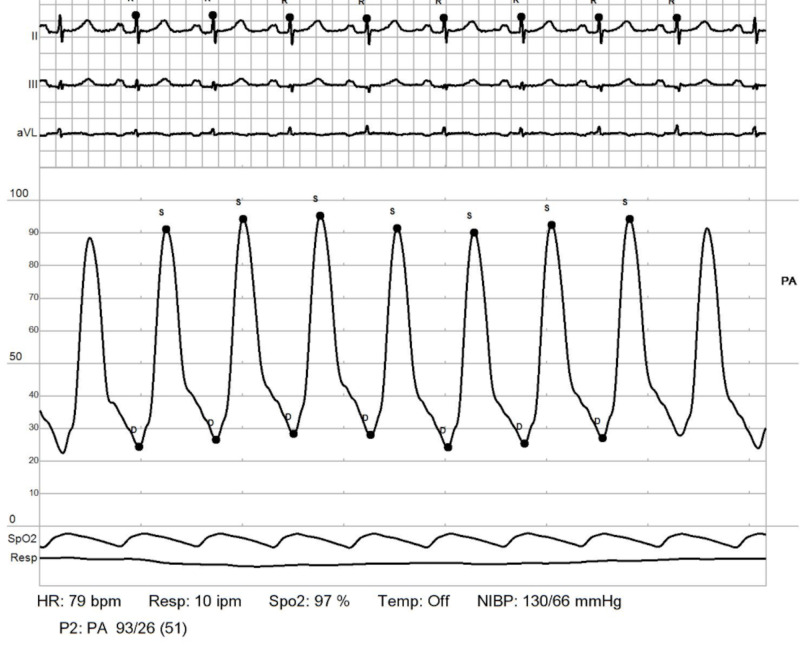
RHC: pulmonary artery. Showing elevated elevated pulmonary artery pressures (PAPs) with mean PAP: 51 mmHg. Transpulmonary pressure gradient (TPG) = 51-11 = 40 mmHg RHC: right heart catheterization

**Figure 10:**
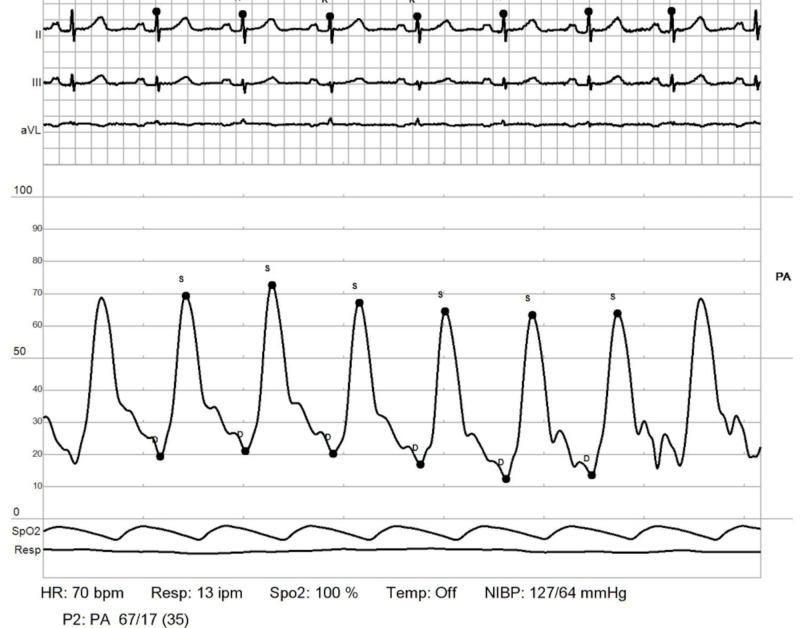
RHC: pulmonary artery - post reactivity. Positive post reactivity test consistent with drop in mean pulmonary artery pressure (PAP) from 51 to 35 mmHg (drop of >10 mmHg or pressure <49 mmHg) RHC: right heart catheterization

Due to further rapid decline in clinical status over the next two to three months, she required inpatient care with aggressive diuresis and empiric treatment for possible pneumonia. She continued to be significantly hypoxic with desaturations to 70% on room air raising concern for an acute flare of underlying ILD as a precipitating event. Repeat TTE showed pulmonary artery systolic pressure worsened to 87 mmHg with RV dilation which had increased from 59 mmHg within one year. Repeat CT chest remained consistent with diffuse septal thickening in the setting of chronic interstitial disease. With continued increment in oxygen requirement, PFTs and CT findings were out of proportion to the degree of pulmonary hypertension which warranted a RHC where her hemodynamics was significant for elevated PAP of 96/28 mmHg (mean 51), pulmonary capillary wedge pressure (PCWP) 11 mmHg, and peripheral vascular resistance (PVR) of 9.6 Woods Units. The nitric oxide vasoreactivity test was positive demonstrating a drop in her mean PAP from 51 to 35 mmHg with CO (cardiac output)/CI (cardiac index) 4.3/2.4 (pulmonary reactivity criteria: fall in mean PAP to <49 mmHg or drop of at least 10 mmHg, or maintenance/increase in cardiac output) [3] (Figures 6-10).

**Figure 11:**
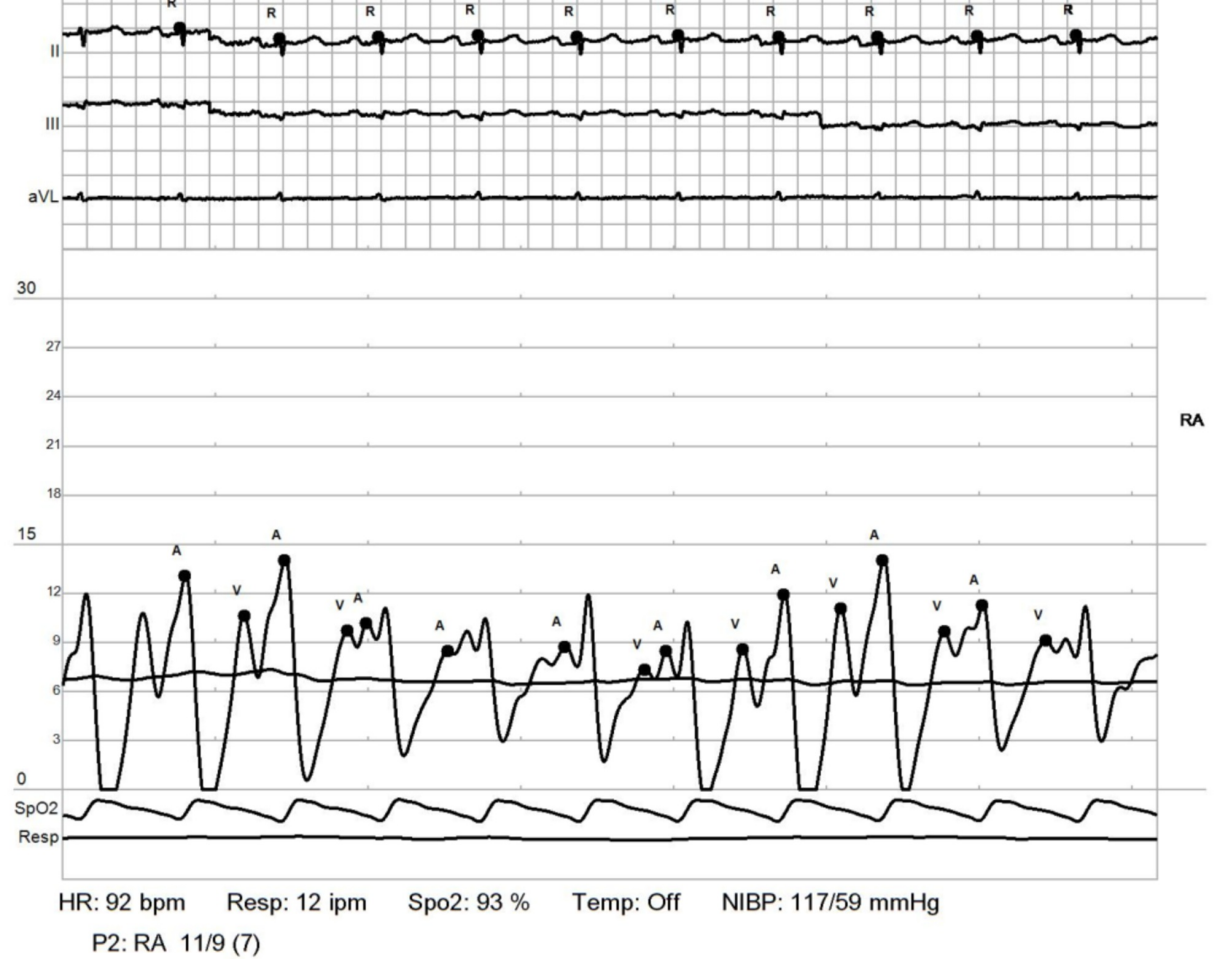
Repeat RHC: right atrium. RHC: right heart catheterization

**Figure 12:**
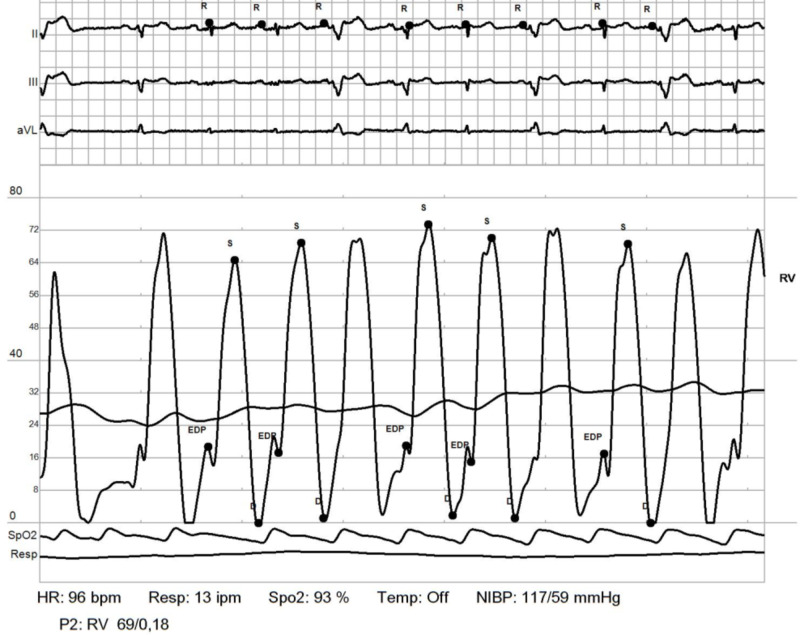
Repeat RHC: RV. RHC: right heart catheterization; RV: right ventricle

**Figure 13:**
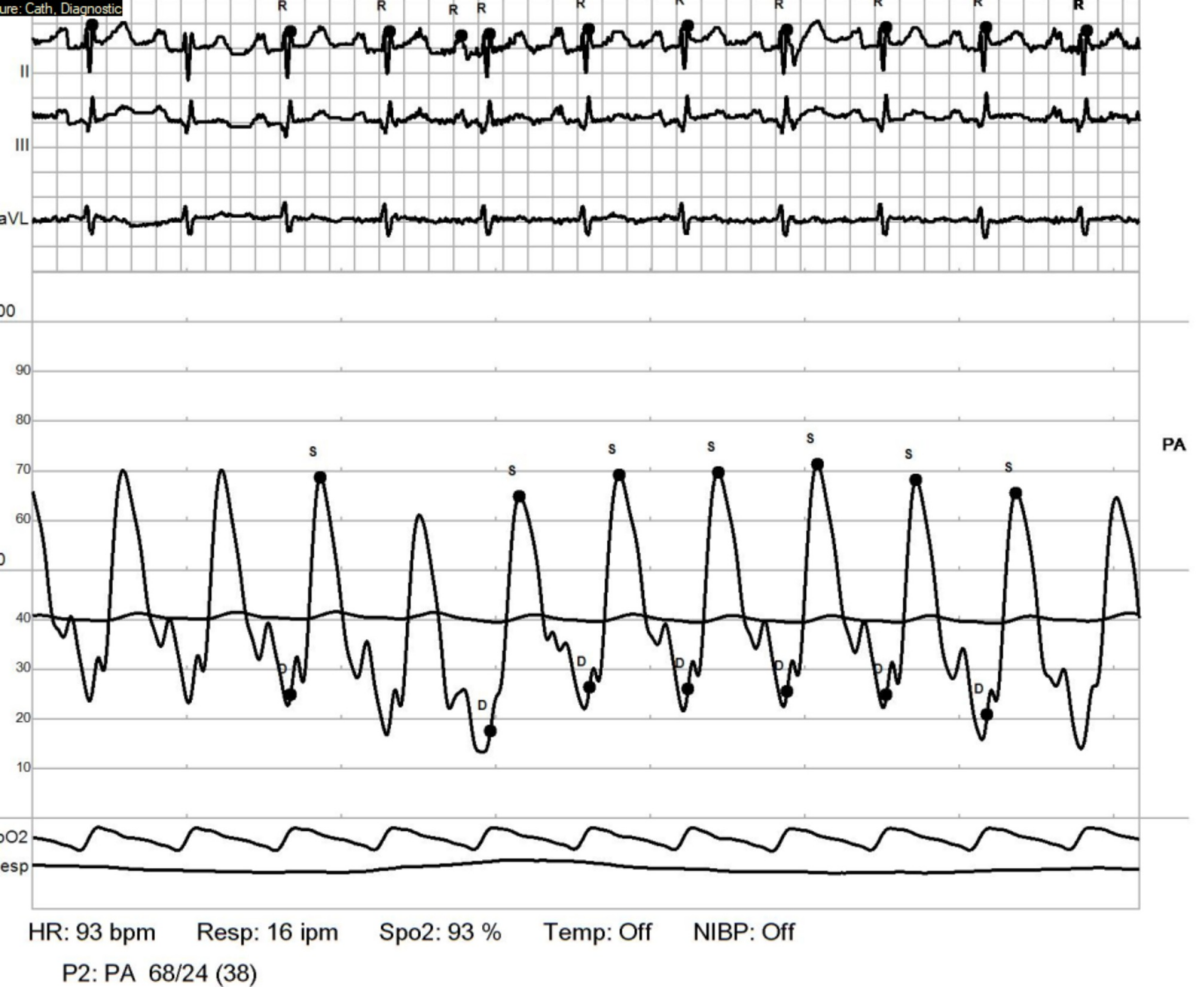
Repeat RHC: PCWP. RHC: right heart catheterization; PCWP: pulmonary capillary wedge pressure

**Figure 14:**
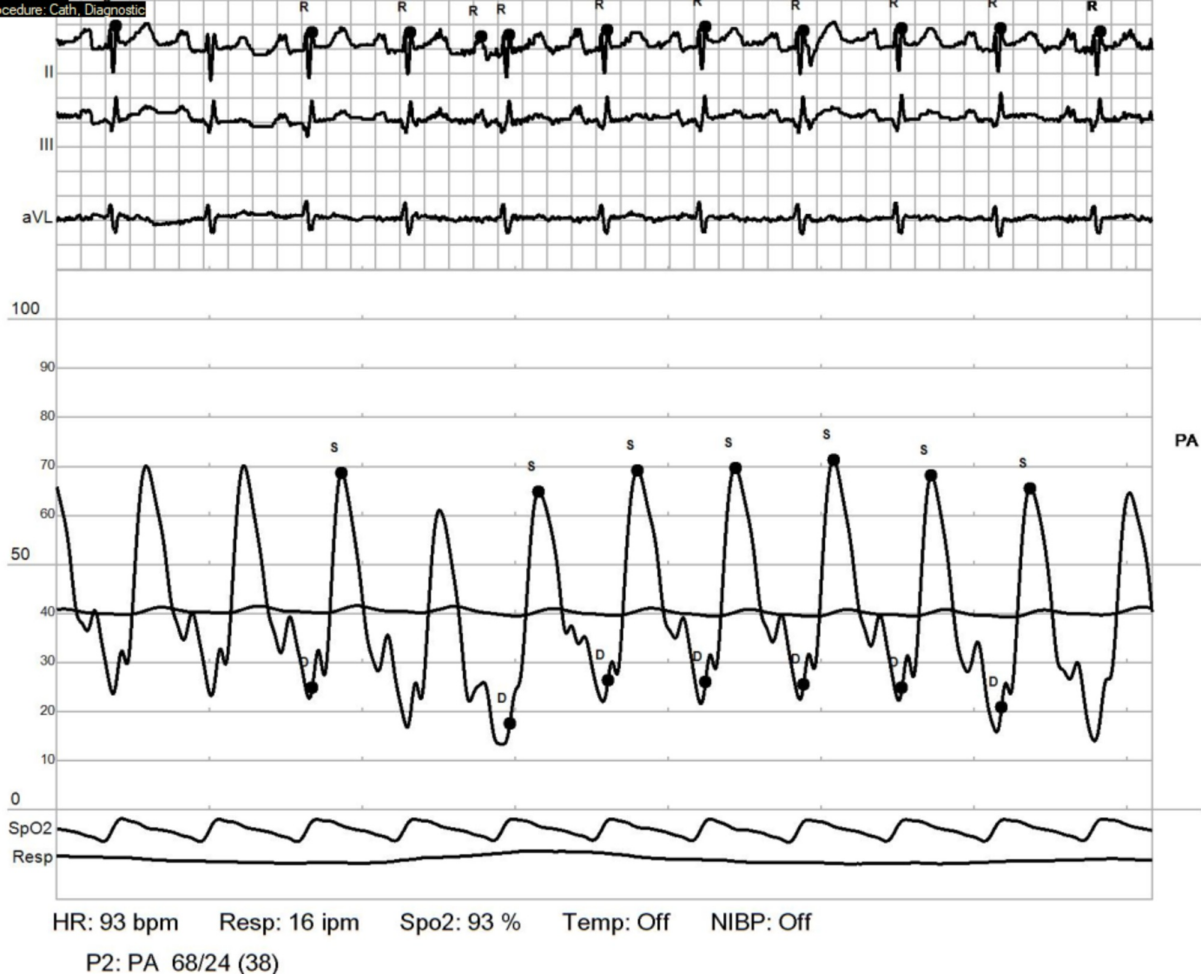
Repeat RHC: pulmonary artery. RHC: right heart catheterization

Therefore, she was started on nifedipine to be uptitrated clinically. Following a multi-speciality pulmonary hypertension conference with pulmonology, cardiology and rheumatology recommendations, she was started on triple therapy: prostacyclin receptor agonist - selexipag, endothelia receptor antagonist - macitentan, and tadalafil. She also continues to be on nifedipine, torasemide, steroids, and mycophenolate. She underwent a repeat RHC after six months interval with hemodynamics showing, PAP 72/24 mmHg, PCWP 18 mmHg, PVR 3.5 Woods Unit, findings consistent with mild improvement in PVR while she continues to be optimized on medical management as titration of above (Figures 11-14).​​​​​​​

## Discussion

Pulmonary hypertension often presents as a multifactorial entity, although an underlying inciting event may be the primary factor unmasking multifactorial nature of pulmonary hypertension [1-3]. PAH may be idiopathic, heritable or associated with connective tissue disease, HIV, congenital heart disease, portal hypertension or drugs. Whereas, secondary pulmonary hypertension is post-capillary or venous in origin. This includes cardiac, pulmonary, thromboembolic and others such as hemolytic disorders, systemic conditions like sarcoid, pulmonary histiocytosis, lymphangioleiomyomatosis, metabolic disorders or chronic renal failure among others [3-5]. We present the case of a 77-year-old female with debilitating dyspnea in the setting of ILD, obstructive lung disease, heart failure with preserved ejection fraction, sleep apnea, chronic anemia secondary to MDS, and newly diagnosed SSc. 

The patient’s pulmonary hypertension was initially considered secondary to ILD, OSA, heart failure, and MDS. Due to her rapidly worsening symptoms being out of proportion to the clinical evidence of precipitating etiology, due to clinical suspicion she led to an extensive workup resulting in diagnosis of SSc. Most common pulmonary effects of SSc include pulmonary vascular disease and ILD. Therefore, SSc can lead to WHO group 1 PAH, or group 3 pulmonary hypertension due to ILD [3-7]. PAH remains the most common cause of pulmonary hypertension in SSc with a prevalence of around 10%-15%. Effects of ILD is present in earlier stages, although usually after the diagnosis of SSC. Development of severe pulmonary hypertension and ILD leading to diagnosis of SSc in this case is unusual. Effects of ILD present in earlier stages of SSc, however, development of pulmonary symptoms prior to diagnosis of SSc is also less likely. Which makes this presentation unusual by development of ILD and pulmonary hypertension leading to the diagnosis of SSc. Long standing SSc and presence of anti-centromere antibody have more likelihood for developing PAH. Due to the unusual acceleration of symptoms despite optimized therapy for multifactorial causes and in light of RHC findings in our patient, PAH is considered to be the primary etiology. Hence, PAH secondary to SSc in this patient was exacerbated by ILD also secondary to SSc, heart failure, sleep apnea along with chronic anemia due to MDS. 

In addition to diagnosis, management of such a case remains a challenge, with concern for optimal focus towards likely all precipitating causes. The patient was started on oral therapy with calcium channel blockers for vasoreactive PAH, along with disease modifying drugs for SSc with mycophenolate, steroids, prostacyclin receptor agonist, endothelin receptor antagonist, diuretics, steroids, reinforcing compliance with CPAP for OSA, optimization of medications for heart failure, and continuing treatment for anemia and MDS. Such multifactorial cases with PAH, should also focus on management of co-morbidities with a multidisciplinary team approach to optimize management from every aspect of contributing factors [5-8].

## Conclusions

The cause of rapid clinical decline in pulmonary hypertension may be its unidentified multifactorial nature. It is imperative to revisit possible etiologies in such a case with extensive and relevant investigations. Despite evident causes, due consideration should be given to undiagnosed autoimmune disorders playing a role in causing PAH. Management in such cases remains a multidisciplinary approach.
